# Sleepless Nights

**Published:** 1874-06

**Authors:** 


### SLEEPLESS NIGHTS.

AN inability to sleep well at night is so common, and arises from such a variety of causes, reflecting persons will direct their attention to two things: Let each one ascertain for himself the cause of his unrest, and remove that cause. This is infinitely more philosophical than to take chloral hydrate, opium or any form of anodyne whatever. If late and hearty suppers cause restlessness, make the last meal of the day of cold bread and butter and a cup of hot drink, and nothing else whatever; try this a week, note the result and act accordingly.

Some do not sleep well, because the head is so low that the blood flows toward it by gravitation, and does not get back easily, hence, it accumulates in the veins, makes the head too full and sleep is impossible.

If the brain is excited at bed time from study, passion or solicitude, then too much blood is carried to it by the arteries, and refreshing sleep is equally impossible. There are those who toss and tumble by the hour, the mind running round in the same circle of ruinous thought, producing in some cases an agony of sweat in great drops on the forehead. It is most mischievous for such persons to remain in bed a single moment; a thousand times better get up and wash and dress, midnight though it be, and either take a walk or indulge in light reading, or some deeply interesting narrative until you get sleepy, then slide into bed as easily as possible.

It often happens that a person wakes up in the night from some unusual circumstance, and is not able to go to sleep for some time afterward; then there is a tendency to wake up at the same time next night without cause, and before one knows it it has become a habit; it can be broken up entirely in forty-eight hours thus: Go to bed two hours later and be waked up an hour earlier; this is an almost infallible remedy. Hunger, or cold feet, or excessive weariness may prevent sound sleep. Persons who do not sleep well should eat moderately of plain, nourishing food, not tempt the appetite; this aggravates the disease by making too much blood; on the other hand if too little is eaten the nerve tissues are starved and make too little hydro-carbon; neither leave off study altogether, nor exercise too much.

